# Increased Parasitic Load in Captive-Released European Bison (*Bison bonasus*) has Important Implications for Reintroduction Programs

**DOI:** 10.1007/s10393-018-1327-4

**Published:** 2018-03-16

**Authors:** Marta Kołodziej-Sobocińska, Aleksadner W. Demiaszkiewicz, Anna M. Pyziel, Rafał Kowalczyk

**Affiliations:** 1 Mammal Research Institute, Polish Academy of Sciences, Stoczek 1, 17-230 Białowieża, Poland; 2 W. Stefański Institute of Parasitology, Polish Academy of Sciences, Warsaw, Poland

**Keywords:** Blood-sucking nematode, *Ashworthius sidemi*, Captive breeding, Conservation management, Immune response

## Abstract

Captive-bred animals, widely used in reintroduction programmes, are often immunologically naïve and more susceptible to pathogens. We analysed infection of invasive blood-sucking nematode *Ashworthius sidemi* in captive-bred European bison (*Bison bonasus*) released to the wild in the Białowieża Forest (Poland). Mean *A. sidemi* infection intensity of released bison (29,137 nematodes) was over threefold higher than in wild bison (8756). It indicates a rapid acquisition and increase in the infection intensity in previously dewormed bison released from captivity. Thus, reintroduction programmes should consider the impact of pathogens and involve controlled exposure of captive animals to specific parasites prior to release.

Diseases play an important role in wild populations, strongly influencing their condition and demography (Scott 1988). They are often a real threat to rare and endangered species, whose survival is one of the main issues in conservation programmes (Pucek et al. 2004; Viggers et al. 1993). Captive-bred animals are often used in reintroduction programmes (Mathews et al. 2006; Tear et al. 1993), especially in rare species for which captive breeding is the most important source of individuals for release (Tear et al. 1993). Captive animals are regularly dewormed to prevent spread of parasitic diseases, and in most cases they have no prior contact with pathogens occurring in the wild and are very likely to be immunologically naïve and more susceptible to pathogens present in the environment (Viggers et al. 1993). Host susceptibility to disease is influenced by several factors, including the following, among others: (1) population density (pathogen transmission, nutritional and social stress), (2) innate resistance and the ability of the host to mount an immune response and (3) the stress associated with reintroduction, which may increase pathological effects of infectious agents and further debilitate released animals (Viggers et al. 1993; Wakelin 1978). Thus, the importance of diseases and their impact on reintroduced animals should be taken into account in conservation and reintroduction programmes.

The European bison (*Bison bonasus* L., 1758) was extirpated in the wild at the beginning of twentieth century and later restored with captive survivors. There are now over 4000 European bison in the wild distributed between 35 isolated populations (Raczyński 2015). Additionally, 2000 bison live in numerous breeding centres and zoos, which constitute an important source of individuals for reintroductions (Raczyński 2015). Bison are one of the main species in rewilding programmes in Europe, and there have been discussions on the need to establish new herds and create a more continuous distribution range (Kuemmerle et al. 2011). Apart from small herd size, isolation and low genetic variation due to a severe genetic bottleneck after extinction in the wild (Tokarska et al. 2011), diseases and parasites (Pucek et al. 2004) are the main threats to bison. Among the 88 parasite species discovered in European bison (Dróżdż et al. 1998; Karbowiak 2014a, b), the blood-sucking nematode *Ashworthius sidemi* is probably the most pathogenic. This parasite is specific to Asian deer species, especially sika deer (*Cervus nippon*) (Dróżdż et al. 1998) and was first found in bison from Białowieża Forest (BF) in 2000 (Kołodziej-Sobocińska et al. 2016a). Adult nematodes are located in abomasa of bison and other ruminants; eggs are shed with faeces. Bison get infected usually directly by ingestion of contaminated food (Dróżdż et al. 1998). Previous studies have shown the influence of herd size and winter bison aggregation on *A. sidemi* and coccidia infection (Kołodziej-Sobocińska et al. 2016a; Pyziel et al. 2011; Radwan et al. 2010) as well as on the seasonal pattern of parasite egg excretion (Kołodziej-Sobocińska et al. 2016c).

In this study, we investigated the parasitic load of five captive-bred bison released into the wild and then culled after some time, compared with simultaneously studied 41 wild-born animals. We hypothesised that captive-bred bison are more susceptible to invasion because they have not acquired immunity to *A. sidemi* while living in captivity.

The study was conducted in BF, located on the Polish–Belarusian border, which is inhabited by the largest free-living bison population (over 500 bison in the Polish part of the forest). Bison is fully protected and endangered species. The bison population in BF has been regulated since the 1970s through culling or translocation (Krasińska and Krasiński 2013). Between 2002 and 2016, 57 culled bison males over 1 year old, including five bison released from captivity, 11 captive and 41 wild-born and free-living individuals, were parasitologically examined postmortem. Limited number of individuals released from captivity results from rarity of studied species, for which collection of larger samples is very difficult. The five bison released from captivity as subadults spent between 179 and 1485 days (609 ± 569 on average) in the wild (Table 1) and were culled by Białowieża National Park (BNP) staff. As all the released bison were culled due to necrotic disease of the external genital organs (posthitis) (Jakob et al. 2000), the comparative group of wild bison also included males culled due to this disease. The entire contents of the abomasa of culled bison were examined according to Dróżdż et al. (1998). Nematode species were determined morphologically (Dróżdż et al. 1998), and the number of *A. sidemi* was counted.

**Table 1:** Data of Bison Males Released from Captivity into the Wild in the Białowieża Primeval Forest, the Length of Their stay in the Wild and the Blood-Sucking Nematode *A. sidemi* Infection Intensity.

Bison pedigree number	Age (months)	Weight (kg)	Date of birth and culling	Date of the release	No. of days spent in the wild	*A. sidemi* number
9538	47	470	26th April 2001–9th March 2005	27th August 2004	194	23,790
9541	45	460	12th May 2001–22nd February 2005	27th August 2004	179	5504
10,772	30	280	25th June 2006–16th December 2008	19th February 2008	301	32,940
10,773	66	560	18th September 2006–14th March 2012	19th February 2008	1485	5820
11,010	69	370	22nd May 2005–2nd February 2011	1st September 2008	884	77,630

Bison released from captivity had from 5504 to 77,630 *A. sidemi* nematodes per bison (Table 1); 29,137 ± 13,222 on average. Their infection intensity was significant and over threefold higher than in wild bison (mean 8756 ± 1418; range 0–37,670; *p* = 0.048; *U* = 46.0), and over 2000 times higher than in captive bison (mean 14.0 ± 7.0; range 0–67; *p* = 0.0016; *U* = 0.0) (Fig. 1). The data suggest that bison released from captivity into the wild suffer a rapid increase in *A. sidemi* infection intensity.

**Fig. 1:**
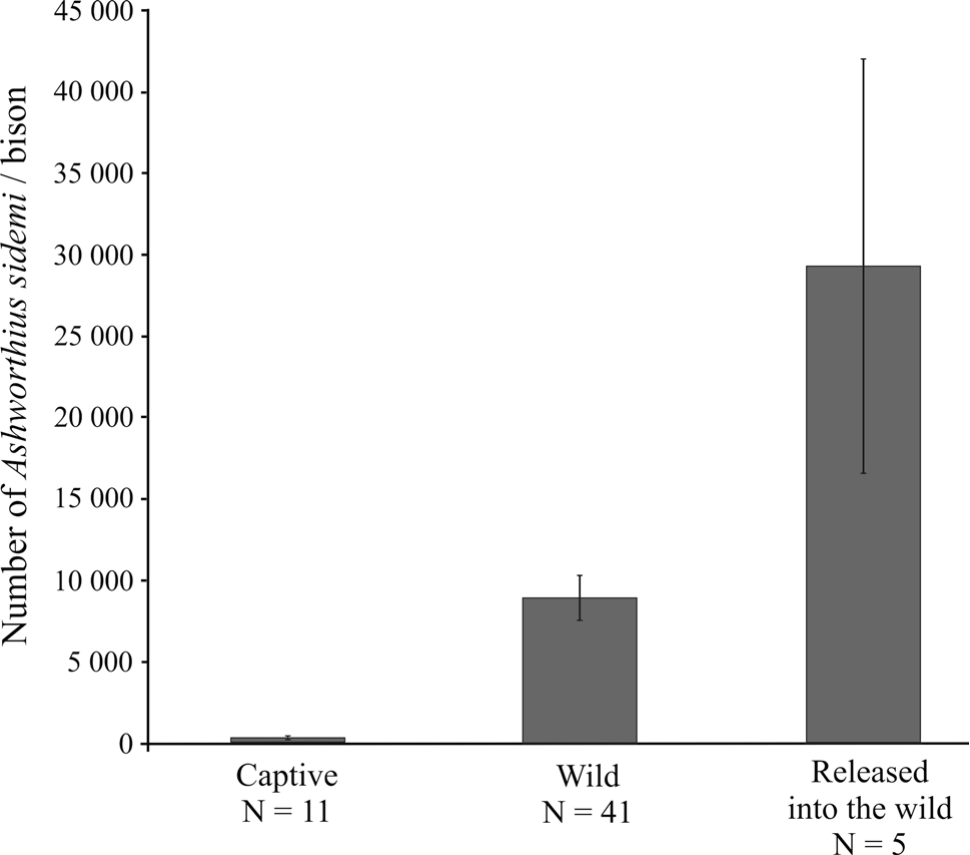
Comparison of *A. sidemi* infection intensity in three European bison male groups in Białowieża Forest: captive, wild and released from captivity into the wild.

Captive breeding programmes are often source of animals for release to supplement or re-establish endangered species populations to prevent their extinction, enrich their gene pool or establish new populations in the wild (Daszak et al. 2000; Faria et al. 2010). Factors that are taken into account in reintroduction programmes include usually: habitat suitability, animal behaviour and density, predators and competitors, the genetic issues, and considerable financial and human support to implement and monitor the programme for many years (Viggers et al. 1993). However, diseases and parasites can also play an important role (Cunningham 1996; Daszak et al. 2000; Schmidt-Posthaus et al. 2002; Viggers et al. 1993). This is because the reduced exposure to natural antigens and loss of immunogenetic variation during captive breeding may impair the survival probability of released animals (Cunningham 1996; Mathews et al. 2006; Viggers et al. 1993). Thus, the risk of diseases and mentioned above associated factors should be considered in the conservation programmes, especially for rare and endangered species. In our study, the animals released from captivity quickly gained a high parasitic load, much higher than wild animals of similar sex and age. This indicates high susceptibility of captive-bred and immunologically naïve individuals to parasitological infection (Cunningham 1996). A high infection level of *A. sidemi* leads to deterioration of red blood cell parameters, which may weaken bison and increase their susceptibility to other pathogens (Kołodziej-Sobocińska et al. 2016b). As many natural populations tend to be held in long-term balance by host–pathogen interactions (May 1988), acquisition of immunity to some diseases may require long-term and low-intensity exposure to pathogens (Wakelin 1978). Moreover, host susceptibility to disease may be influenced by population density which might increase pathogen transmission and social stress, innate resistance and the ability to mount an immune response (Radwan et al. 2010; Wakelin 1978). For example, the increased contact rate between bison in winter due to large aggregations arising from supplementary feeding in fixed locations promotes parasite transmission (Karbowiak et al. 2014b; Kołodziej-Sobocińska et al. 2016a; Pyziel et al. 2011; Radwan et al. 2010). The five bison released from captivity stayed in the places where supplementary feeding was intense, which further increased the number of parasites transmitted to these immunologically naïve individuals. Higher *A. sidemi* infection intensities were associated with longer stays of released bison in the wild, which suggests bison suffered repeat infections and insufficient immune responses.

Bison in captivity are regularly dewormed (Krzysiak et al. 2015), which is a standard procedure to avoid parasite transmission in captivity. However, treatment of animals prior to release may not be beneficial, because it may reduce their levels of immunity to disease (Faria et al. 2010; Viggers et al. 1993). This is especially important if such pathogens are present in wild populations, as is the case of blood-sucking nematode *A. sidemi* in the bison population in BF (Dróżdż et al. 1998; Kołodziej-Sobocińska et al. 2016a, c); regularly dewormed captive bison have no chance to acquire immunity against *A. sidemi* or other parasites that they come into contact with upon release. Moreover, release into the wild probably stresses bison, which could make them more susceptible to infection (Dickens et al. 2010). It has been proposed that when there are parasites present in wild populations to which captive animals have not been exposed, then it may be advisable to provide low-level exposure in captivity to develop some immunity (Viggers et al. 1993). Recently, since 2012, low *A. sidemi* infection intensity has been reported in captive bison (Kołodziej-Sobocińska et al. 2016b; Krzysiak et al. 2015). Hopefully, controlled exposure of animals to parasites prior to release may be beneficial and increase their immunity.

The impacts of disease have been neglected in mammal reintroduction programmes, including those involving European bison. Thus, the management protocols should be established and standardised for endangered species reintroductions. These would help to avoid hazards and prevent failure of the programmes caused by exposure to new diseases and weakening of naïve individuals released from captivity. In recent years, European bison have been introduced to new places in Europe; by understanding the factors that can cause failure of bison reintroductions, we can design more optimal conservation management approaches. This would help to reduce the conservation risk though exposure of naïve captive animals to pathogens present in the wild.
